# Reference values for Jamar+ digital dynamometer hand grip strength in healthy adults and in adults with non-communicable diseases or osteoarthritis: the Norwegian Tromsø study 2015–2016

**DOI:** 10.1007/s10433-023-00791-w

**Published:** 2023-11-24

**Authors:** Odd-Einar Svinøy, Gunvor Hilde, Astrid Bergland, Bjørn Heine Strand

**Affiliations:** 1 Faculty of Health Sciences, Department of Rehabilitation Science and Health Technology, OsloMet – Oslo Metropolitan University, Oslo, Norway; 2 Department of Physical Health and Ageing, Norwegian Institute of Public Health, Oslo, Norway; 3 Norwegian National Centre for Ageing and Health, Vestfold Hospital Trust, Tønsberg, Norway; 4 Department of Geriatric Medicine, Oslo University Hospital, Oslo, Norway

**Keywords:** Hand grip strength, Aging, Osteoarthritis, Non-communicable diseases (NCD), Cross-sectional studies, Epidemiology

## Abstract

Hand grip strength (HGS) is a key indicator of intrinsic capacity and has shown good predictive ability for morbidity and mortality. Reference values from normative populations are valuable, and such data from the Norwegian population are scarce. Normative values for the digital Jamar+ dynamometer are largely lacking.HGS was assessed in the Norwegian Tromsø study, survey 7 in 2015–2016 for 7824 participants (9324 invited) aged 40+ using a Jamar+ digital dynamometer, and three measurements for each hand were performed following the Southampton protocol. To account for non-response, full Tromsø population data, by age, education and sex, were collected from registry data from microdata.no, a service from Statistics Norway, and were then used as post-stratification weights, to provide standardized HGS values. HGS was higher in men than in women and inversely associated with age. Men and women with a history of non-communicable diseases had lower HGS than those without these conditions, while osteoarthritis was associated with lower HGS only among men. Lower height was associated with lower HGS, especially at younger ages in men. This article provides up-to-date references values for HGS in the community-dwelling population aged 40+ with or without osteoarthritis or non-communicable diseases, in Tromsø, Norway. These reference values will guide clinicians and researchers.

## Background

Various normative hand grip strength (HGS) values have been published for the hydraulic Jamar dynamometer, while normative values for the digital Jamar+ dynamometer are lacking (Peters et al. 2011). HGS (Hamilton et al. 1994) is a commonly used test of physical function, and it is a good indicator of overall muscular strength (Bohannon 2008), as well as a key domain of intrinsic capacity (WHO 2015, 2021). The decrease of muscular strength with aging is well documented (Landers et al. 2001; Sternfeld et al. 2002), and reduced HGS is associated with sarcopenia and frailty (Fried et al. 2001; Mijnarends et al. 2013; Sayer et al. 2013; Syddall et al. 2003). As HGS weakens with age and disease onset, older adults are more likely to have trouble with daily activities implying loss of independence (Norman et al. 2011). Reduced muscle strength is associated with a wide range of health and health-related outcomes, such as increased postoperative complications, increased length of hospitalization, higher rehospitalization rate, increased likelihood for future hospitalizations, decreased physical status, multiple chronic diseases compared and lowered health-related quality of life (Allard et al. 2016; Cheung et al. 2013; Humphreys et al. 2002; Hunt et al. 1985; Norman et al. 2011; Sayer et al. 2006; Simmonds et al. 2015). HGS has been highlighted as an indispensable stand-alone biomarker for older adults (Bohannon 2019a). A decline in HGS is correlated with onset of morbidity and reduced survival (Gale et al. 2007). It is reported that for each standard deviation (SD) increase in HGS the relative risk of cardiovascular disease and all-causes mortality is reduced (Gale et al. 2007). Earlier in the Tromsø study, we found that for each SD reduction in HGS the relative risk of mortality increased by 17 percent (Strand et al. 2016). Also, those with low HGS are more likely having multiple chronic diseases compared to those with high HGS (Cheung et al. 2013). Recent research has also shown that low HGS is associated with joint space narrowing in subjects with hand osteoarthritis and also among subjects with knee osteoarthritis (Wen et al. 2017). Due to its simplicity and predictive abilities, HGS testing has been suggested for standard routine use for vital signs, nutritional status and as a screening tool (Bohannon 2008; Klidjian et al. 1980; Lee et al. 2017; Norman et al. 2011), making early risk identification and intervention feasible (Giampaoli et al. 1999). As such, there is growing interest in its assessment in clinical settings. Measurements of HGS are simple to perform in a clinical setting, non-invasive and inexpensive, making it readily available for clinicians. HGS can be measured in several ways, but the Jamar dynamometer is the most widely used instrument in research and is suggested to be the gold standard among dynamometers (Roberts et al. 2011), and is also recommended in a recent European consensus (Cruz-Jentoft et al. 2019). Hand dominance does not appear to be significant when it comes to HGS (Günther et al. 2008).

Currently published data on normative values for HGS are available for the healthy population and general population from different countries (Amaral et al. 2019; Ekşioğlu 2016; Kim et al. 2018a, b; Kim et al. 2018a, b; Lam et al. 2016; Malhotra et al. 2016; Mat Jais et al. 2018; Ong et al. 2017; Steiber 2016; Wang et al. 2018; Wearing et al. 2018; Wong 2016; Yoo et al. 2017; Yu et al. 2017) in addition to several meta- and pooled analyses (Dodds et al. 2014; Kamide et al. 2015; Lera et al. 2018). As far as the authors are aware, no previous studies have provided normative values on HGS in patients with osteoarthritis and only one study has provided reference values on HGS in participants with specific chronic diseases (Yorke et al. 2015). Only one study has provided reference values for the general population of Norway, stratified by age and sex (Tveter et al. 2014), but the sample size for each age group was low. The aim of this study was to present up-to-date reference values for HGS for community-dwelling individuals aged 40+ years, with or without osteoarthritis or non-communicable diseases, in Tromsø, Norway.

## Methods

### The Tromsø study

The Tromsø study is a multipurpose population-based study, which was initiated in 1974. Since then, the study has had additional waves in 1979–1980 (Tromsø2), 1986–1987 (Tromsø3), 1994–1995 (Tromsø4), 2001 (Tromsø5), 2007–2008 (Tromsø6) and most recently 2015–2016 (Tromsø7). The data for the current study were based on Tromsø7. Tromsø is the largest city in the Northern part of Norway with 73,480 inhabitants at the time of Tromsø7, predominantly of Norwegian origin (SSB 2021).

### HGS testing procedure in Tromsø7

HGS was assessed by trained health professionals, using a Jamar+ digital dynamometer with a standardized protocol. Participants were asked to sit in a chair holding the dynamometer and resting the arm at the chair’s armrest, 90 degrees angle at elbow, and hand as far as it was free from the armrest, thumb up. The dynamometer’s position two (counted from the display) of five possible settings was used for all participants. Three measurements for each hand were collected, in total six, alternating between right and left hand. In the current study, the maximum value of the six was used, following the Southampton protocol (Roberts et al. 2011). For those with missing values, the maximum value of the performed HGS trials was used.

### Study population

In Tromsø7, all Tromsø inhabitants 40 years and above (*n* = 32,591) were invited for phase-one study, and a random set was invited to take part in the second phase that included comprehensive clinical examinations, including testing of HGS. Out of the 9324 invited to HGS testing, 7824 had at least one measurement recorded. Among these participants, 7701 had no missing values, while 10, 12 and 40 participants had 1, 2 and 3 missing values, respectively, for the left hand, and 5, 6 and 50 had 1, 2 or 3 missing values, respectively, for the right hand. In total, we had 7824 respondents with valid HGS measures (3558 men and 4266 women), which comprised our study population.

### Educational level, height and disease history

Educational level was self-reported and grouped as compulsory, secondary and tertiary. Those with missing value were imputed with compulsory education (181 of 7824). Body height was measured by trained personnel and dichotomized at mean height for men and women when assessing its impact in HGS. For our HGS reference values, we stratified on disease status and sex. We used self-reported disease history for osteoarthritis, cardiovascular disease (CVD: heart attack, heart failure, angina pectoris, cerebral stroke/brain hemorrhage), cancer, pulmonary disease (chronic bronchitis, emphysema) and diabetes. Further, the disease status of osteoarthritis and the four man NCDs were, respectively, dichotomized and coded “yes” if present and coded “no” if not present. Finally, a variable “healthy” was created and coded “yes” if neither NCD nor osteoarthritis was present and “yes” if at least one of the diseases was present.

### Statistical methods

Stata 17 was used for all analyses. To control for possible selection bias due to higher educational level in our study population compared to Tromsø at large, the corresponding educational level (compulsory, secondary and tertiary) in Tromsø at large on January 1, 2016, sex and 5-year age groups (40–44, …, 80–84) were collected through registry data from Microdata.no, a service from Statistics Norway. Standardized by age in 5-year age groups, by the direct method and Tromsø per January 1, 2016, as standard population, the prevalence of tertiary education in our study population in Tromsø7 among men was 50% compared to 44% in Tromsø at large. In women, the prevalence was 48% versus 37% in Tromsø. Based on this, we calculated participation weights (inverse probability weights, IPW) for each sex–age–education stratum, which ranged from 1 to 19. Weights above 10 were set to 10 (7%), to reduce the large influence of the largest weights. The Tromsø population of January 1, 2016, by age, education and sex, was then used as post-stratification weights, to provide standardized mean, SDs and SEs. In addition, percentiles (10th, 25th, 50th, 75th, 90th) were predicted from an IPW-weighted quantile regression model, with age (5-year groups) and sex as dependent variables (and disease status). All three- and two-way interaction terms between covariates were included to allow for full flexibility. In an additional analysis, mean HGS with corresponding 95% confidence interval was estimated from a linear regression model using age as a restricted cubic spline with four knots at default knot location (60, 66, 71, 80 years) and the Tromsø population as post-stratification weights. Sex- and disease-specific normative HGS values were then predicted post hoc from the fitted regression models.

## Results

Among the 7824 participants, 54% were women, and the mean age was 63.1 years (SD 10.4, range 40–84 years), and the interquartile range was 57–71 years (Table 1). Age was similar for men and women. Average height in men was 177 cm and in women 163 cm. More women than men were affected with osteoarthritis (31% vs 15%), while men had higher CVD prevalence (16% vs 7%) and NCD prevalence (31% vs 23%).

**Table 1:** Background characteristics (*N* = 7824)

	Men (*n* = 3558)	Women (*n* = 4266)	Total (*n* = 7824)
Age, mean (SD)	63.0 (10.5)	63.2 (10.4)	63.1 (10.4)
*Age, n (%)*			
40–44	217 (6.1)	278 (6.5)	495 (6.3)
45–49	263 (7.3)	307 (7.2)	570 (7.3)
50–54	250 (7.0)	332 (7.8)	582 (7.4)
55–59	328 (9.2)	437 (10.2)	765 (9.8)
60–64	726 (20.4)	811 (19.0)	1537 (19.6)
65–69	749 (21.1)	855 (20.0)	1604 (20.5)
70–74	528 (14.8)	666 (20.0)	1194 (15.3)
75–79	331 (9.3)	386 (9.1)	717 (9.2)
80–84	166 (4.7)	194 (4.6)	360 (4.4)
*Education, n (%)*			
Compulsory	983 (28)	1447 (34)	2430 (31)
Secondary	1028 (29)	1104 (26)	2132 (27)
Tertiary	1547 (43)	1715 (40)	3262 (42)
Mean height (SD), cm	176.9 (6.8)	163.4 (6.4)	169.6 (9.4)
*Diseases, n (%)*			
NCD^a^	1108 (31.1)	998 (23.4)	2106 (26.9)
CVD	571 (16.1)	304 (7.1)	875 (11.2)
Cancer	395 (11.1)	431 (10.1)	826 (10.6)
Pulmonary disorders	135 (3.8)	188 (4.4)	323 (4.1)
Diabetes	257 (7.2)	245 (5.7)	502 (6.4)
Osteoarthritis	516 (14.5)	1309 (30.7)	1825 (23.3)
Not NCD, not osteoarthritis	2137 (60.1)	2352 (55.1)	4489 (57.4)

*SD* standard deviation, *NCD* non-communicable diseases, *CVD* cardiovascular diseases

^a^The participant is defined as having NCD if the participant has at least one of the conditions CVD (heart attack, heart failure, angina pectoris, cerebral stroke/brain hemorrhage), cancer, pulmonary disorders or diabetes. Thus, the sum of the singular diseases does not sum up to NCD as there is some overlap between conditions

**Fig. 1:**
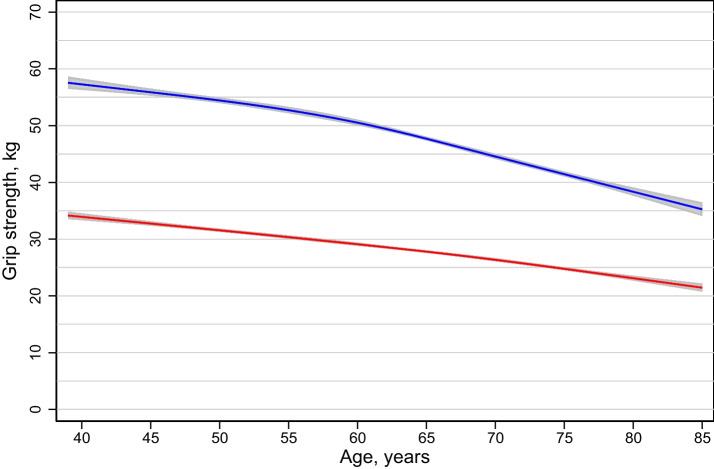
Mean handgrip strength (95% CI) by age and sex (women red and men blue). Scatter plots are actual values. *N* = 7824. Mean and CIs are standardized estimates (standardized to the Tromsø population using post-stratification weights) with age as a cubic spline with four knots

Mean HGS declined with age in both men and women (Fig. 1). In men, HGS was 55.7 kg at ages 40–44 years, 53.1 kg at ages 50–54 years, 48.9 kg at ages 60–64, 43.9 at ages 70–74 and 36.8 at ages 80–84 (Table 2). In women, HGS was 33.3 kg at 40–44 years, 30.8 kg at 50–54 years, 28.7 kg at 60–64 years, 25.7 kg at 70–74 years and 22.7 kg at 80–84 years. The percentiles followed similar downward pattern with age. In men, the interquartile range (25th to 75th percentile, IQR) was 50.0–60.7 kg at age 40–44 years and 32.6–40.7 kg at age 80–84. In women, the corresponding IQRs were 29.6–36.7 kg and 19.7–26.1 kg.

**Table 2:** Hand grip strength (kg) mean (SD) and percentiles (10th, 25th, 50th, 75th and 90th) by age and sex (*N* = 7824)

Age	*n*	Mean	SD	p10	p25	p50	p75	p90
*Men*								
40–44	217	55.7	7.4	43.3	50.0	56.1	60.7	65.8
45–49	263	56.4	7.2	44.8	50.5	56.8	61.8	66.9
50–54	250	53.1	7.8	42.6	48.1	53.4	58.2	63.2
55–59	328	52.2	7.4	41.5	46.8	52.2	57.7	63.1
60–64	726	48.9	7.2	38.9	43.3	48.9	53.8	58.5
65–69	749	46.1	6.9	37.2	40.8	45.9	51.1	56.3
70–74	528	43.9	7.5	34.2	38.3	43.4	49.3	53.9
75–79	331	40.7	6.4	32.0	35.8	40.2	45.2	50.3
80–84	166	36.8	7.6	27.4	32.6	37.2	40.7	45.0
*Women*								
40–44	278	33.3	4.9	26.8	29.6	33.0	36.7	40.1
45–49	307	32.7	4.8	26.3	29.1	32.7	36.1	39.0
50–54	332	30.8	5.0	24.3	27.4	30.7	34.2	37.6
55–59	437	29.7	4.2	23.7	26.5	29.5	33.1	36.0
60–64	811	28.7	4.2	23.1	25.7	28.4	31.7	34.5
65–69	855	27.2	4.4	21.5	24.1	27.1	30.5	32.8
70–74	666	25.7	5.1	19.6	22.9	25.9	28.8	31.5
75–79	386	24.2	4.7	18.9	21.4	24.0	26.9	30.6
80–84	194	22.7	4.5	16.3	19.7	22.7	26.1	28.4

Mean and SD are standardized estimates (standardized to the Tromsø population in 2016 by age, sex and education using post-stratification weights), while percentiles are predicted from inverse probability weighted quantile regression with age group by sex interaction

*SD* standard deviation

Height was positively associated with HGS at all ages, in both men and women (Table 3). Adjusted by age, men below average height (< 176.9 cm) had on average 4.6 kg (95% confidence interval (CI) 4.0, 5.2) lower HGS than those above average height. The difference was more pronounced at younger age groups in men. For women, those below mean height of 163.6 cm had 3.1 kg (95% CI 2.7, 3.4) lower HGS than women above mean height, and this difference was similar across age groups.

**Table 3:** Hand grip strength (kg) mean (SD) and percentiles (10th, 25th, 50th, 75th and 90th) by age, sex and height (*N* = 7824)

Age	*n*	Mean	SD	P10	P25	P50	P75	P90	*n*	Mean	SD	P10	P25	P50	P75	P90
*Men* < *176.9* *cm*									*Men* > *176.9* *cm*							
45–49	82	51.3	7.0	42.2	45.0	51.4	57.6	60.3	135	58.3	7.4	48.2	54.3	58.2	63.3	66.9
50–54	95	53.8	6.7	44.6	47.7	53.2	59.5	63.5	168	57.9	7.8	46.5	53.0	58.0	63.0	68.2
55–59	93	50.8	8.0	41.3	46.5	50.3	56.3	61.4	156	54.6	8.4	46.0	49.6	54.8	58.7	64.9
60–64	121	49.1	7.1	39.0	44.6	49.1	53.9	58.4	207	54.1	8.0	44.6	48.6	53.5	59.3	64.8
65–69	329	45.8	7.5	36.8	40.9	46.3	50.6	53.9	397	51.5	7.1	42.1	47.1	51.7	56.1	60.4
70–74	398	44.3	6.5	36.1	39.6	44.6	48.6	52.9	348	48.2	8.0	38.4	42.0	48.1	54.1	58.2
75–79	303	42.4	7.2	33.3	37.4	42.4	47.4	51.4	225	45.9	8.8	35.1	40.6	46.0	52.2	55.7
80–84	240	39.5	6.5	31.2	35.1	39.4	44.3	47.3	91	43.6	7.4	34.6	39.3	42.7	49.4	52.6
*Women* < *163.6* *cm*									*Women* > *163.6* *cm*							
40–44	99	31.2	4.0	25.6	28.2	30.8	34.9	36.7	178	34.4	5.3	27.6	30.6	34.3	38.2	41.1
45–49	103	30.7	5.3	25.0	27.8	30.7	34.4	36.0	204	33.7	4.5	27.8	30.2	33.8	37.0	39.8
50–54	134	28.8	5.0	23.4	25.5	29.0	32.1	34.5	198	32.1	5.1	25.4	28.9	31.8	35.5	39.5
55–59	181	27.5	4.3	22.0	24.3	27.5	30.7	33.6	254	31.4	4.0	26.1	28.5	31.1	34.3	37.0
60–64	384	27.4	4.2	22.3	24.7	27.3	30.3	32.7	424	29.8	4.5	23.9	26.9	29.5	33.1	35.8
65–69	446	25.5	4.2	20.9	23.1	25.3	27.9	31.0	407	29.0	4.5	23.5	26.3	29.2	32.0	34.0
70–74	394	24.8	5.9	18.8	22.1	24.9	27.6	30.4	270	26.9	4.6	20.8	24.0	26.8	30.0	32.4
75–79	266	23.3	4.6	17.9	21.2	23.3	25.8	28.4	118	26.2	5.3	20.7	22.8	25.7	29.2	32.8
80–84	157	22.2	4.8	16.1	19.1	22.2	26.0	28.0	36	24.7	4.0	19.3	22.2	24.9	27.1	30.1

Mean and SD are standardized estimates (standardized to the Tromsø population in 2016 by age, sex and education using post-stratification weights), while percentiles are predicted inverse probability weighted quantile regression with age group by sex by height interaction

*SD* standard deviation.

**Fig. 2:**
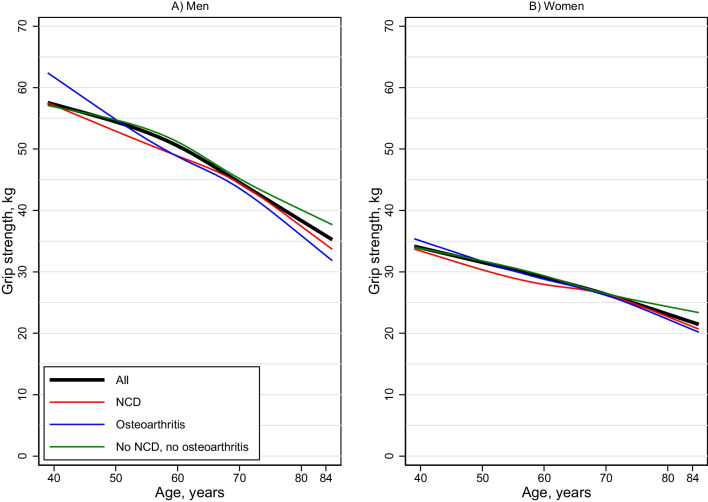
Mean handgrip strength by age sex and disease status. *N* = 7824. Mean grip strength values are standardized to the Tromsø population using post-stratification regression with age as a cubic spline with four knots

Adjusted by age, men with NCD had on average 1.6 kg lower HGS (95% CI 0.9, 2.4) compared with those without NCD (Table 4). In women, the corresponding difference was 0.9 kg (95% CI 0.4, 1.4). The association was similar across age groups in both men and women (no significant interaction of NCD by age groups). Men with osteoarthritis had on average lower HGS than those without this condition, 1.3 kg (95% CI 0.4, 2.3), while in women there was no significant difference; hence, a significant sex interaction was found (*p* < 0.001). Men without NCD and osteoarthritis had on average 1.8 kg higher HGS compared to those with one or both conditions (95% CI 1.1, 2.5) (Table 4, Fig. 2). For women, the corresponding difference was 0.6 kg (95% CI 0.2, 1.0), and also, here we found a significant sex interaction (p < 0.001).

**Table 4:** Hand grip strength (kg) mean (SD) and percentiles (10th, 25th, 50th, 75th and 90th) by age, sex and disease categories

Age	*n*	Mean	SD	P10	P25	P50	P75	P90	*n*	Mean	SD	P10	P25	P50	P75	P90
*Men*									*Women*							
*Osteoarthritis*									*Osteoarthritis*							
40–44	11	59.9	9.5	49.0	49.2	59.7	70.2	70.9	13	34.4	7.8	26.6	29.8	31.4	38.2	46.4
45–49	11	55.5	6.4	46.5	47.2	54.2	60.6	68.6	21	32.7	4.3	27.7	28.6	33.8	35.8	38.8
50–54	22	51.2	8.4	41.9	47.4	49.9	56.3	58.3	60	30.7	6.6	22.5	26.9	30.0	35.0	37.9
55–59	34	51.8	10.2	40.3	44.6	52.3	58.1	66.7	111	30.2	4.8	23.3	27.5	30.7	33.6	35.7
60–64	109	48.0	8.6	38.2	42.9	48.0	53.9	59.6	239	28.1	4.7	22.7	24.8	27.8	31.5	33.9
65–69	110	45.1	7.3	35.2	39.7	45.1	50.3	54.8	317	27.1	5.0	21.3	23.7	27.2	30.5	32.9
70–74	109	41.7	8.9	30.2	36.8	42.1	46.1	53.9	270	25.4	5.7	19.2	23.1	25.7	28.7	31.0
75–79	67	38.7	6.6	29.2	34.3	39.0	43.4	48.4	181	23.3	4.8	17.3	20.6	23.5	25.7	28.4
80–84	43	35.1	7.2	27.5	29.3	36.3	39.0	42.3	97	21.8	4.8	16.1	18.4	22.2	25.2	27.2
*NCD*									*NCD*							
40–44	13	52.3	7.0	42.2	46.9	51.2	56.8	64.1	25	32.3	5.5	25.2	29.4	32.0	34.9	38.5
45–49	23	55.4	10.0	39.8	46.0	56.1	63.0	65.9	34	32.0	5.6	25.0	28.9	32.7	35.8	40.0
50–54	34	50.3	9.2	37.4	46.0	49.5	56.3	59.2	44	28.3	5.7	21.6	25.5	29.4	31.3	34.2
55–59	58	50.1	7.1	39.6	45.0	49.9	55.3	59.8	68	29.1	4.4	23.9	26.1	28.6	32.3	35.5
60–64	193	48.4	7.9	38.3	42.6	48.2	53.9	58.5	151	28.4	4.3	23.2	25.7	28.1	31.4	34.2
65–69	265	45.3	7.5	35.9	39.9	45.7	50.0	54.8	231	27.1	5.0	21.3	23.8	27.2	30.6	33.2
70–74	251	43.7	8.3	33.8	37.5	43.1	49.1	54.3	210	25.1	6.1	19.7	22.3	24.9	28.5	31.2
75–79	181	39.6	7.3	31.7	34.5	39.6	44.4	48.1	142	23.8	5.4	16.9	21.4	23.6	26.8	30.2
80–84	90	36.0	8.3	26.9	32.3	36.3	39.8	43.2	93	22.2	5.0	16.1	19.1	22.2	25.8	28.0
Healthy*									Healthy*							
40–44	194	55.6	7.9	43.3	50.3	56.1	60.7	65.1	241	33.4	5.0	26.8	29.6	33.2	36.8	40.2
45–49	231	56.6	7.5	45.6	50.7	56.9	61.5	66.9	254	32.7	5.1	27.1	29.1	32.7	36.1	39.2
50–54	197	53.8	8.2	44.3	48.3	54.0	59.2	63.5	239	31.2	4.9	24.4	28.0	31.3	34.4	37.7
55–59	242	52.8	7.7	42.2	47.9	52.5	58.0	63.4	282	29.7	4.4	23.8	26.4	29.6	33.2	36.2
60–64	465	49.2	7.5	39.8	44.3	49.1	53.8	58.3	468	28.9	4.4	23.2	25.9	28.6	32.0	35.0
65–69	412	46.8	7.5	37.8	41.3	46.3	51.9	56.8	393	27.2	4.6	22.0	24.1	26.6	30.2	33.0
70–74	221	45.2	7.2	36.7	40.0	44.9	50.3	53.9	276	26.0	5.0	19.9	23.4	26.3	29.3	32.0
75–79	115	42.4	6.4	34.6	38.2	41.8	46.8	51.3	140	25.4	5.2	20.7	21.6	24.5	28.4	32.5
80–84	60	37.9	8.6	28.0	33.6	37.6	42.4	46.7	59	23.9	4.2	17.5	21.2	23.9	27.1	28.9

*SD* standard deviation, *NCD* non-communicable diseases

*Not OA (osteoarthritis) or NCD. Mean and SD are standardized estimates (standardized to the Tromsø population in 2016 by age, sex and education using post-stratification weights), while percentiles are predictions from inverse probability weighted quantile regression with all age group by sex by disease group interactions

## Discussion

This study provides robust, up-to-date Jamar+ digital dynamometer reference values for HGS for a community-dwelling adult population aged 40–84 years in the northern part of Norway. HGS was inversely associated with age and positively associated with body height. Men and women with NCD or osteoarthritis had lower HGS than those without these conditions.

As in the study from the Netherlands by Peters et al. (2011), which applied the same statistical technique as in the current study (quantile regressions with restricted cubic spline functions on age), we found a curvilinear decline with age in men, while in women the decline was linear. As reported by Peters, the decline in men excelled after age 60 years. Our results, however, were slightly higher than those reported by Peters: three to four kg in women and four to five kg in men across all age groups. These differences are probably due to the difference in procedures; Peters used the mean values out of three measurements for each hand, while we used the maximum value of all six, as suggested by the Southampton protocol (Roberts et al. 2011). Alternative explanations for these diverging results may be due to differences in study populations, that we included more recent born birth cohorts, or/and that the Jamar+ digital dynamometer, which we used, may show higher values than the hydraulic version of the Jamar dynamometer for the same applied HGS. Normative values for the digital Jamar+ dynamometer are largely lacking. An exception is the study of 60+ years old in the well-being of the Singapore Elderly study (*n* = 2043) (Ong et al. 2017). This study reports substantially lower HGS than in our study, but data are not directly comparable to our results as the Singaporean study used the mean of two dominant hand grip assessments.

It is assumed that changes of 5.0–6.5 kg may be reasonable estimates of meaningful change in HGS (Bohannon 2019b). Thus, the mean difference of only 1.8 kg in men and 0.6 kg for women for those without NCD and osteoarthritis compared to those with one or both conditions corresponds to about one-fifth of the standard deviation and could be considered minor, even if the difference was significant.

Our findings of low HGS to be associated with increased risk of NCD are in line with previous reports reporting low HGS to be associated with increased risk of cardiovascular disease (Leong et al. 2015), cardiometabolic disorders (CMD) (Hao et al. 2020) and the metabolic syndrome (Sayer et al. 2007). Furthermore, a recent study of older adults in Malaysia found low HGS to be associated with increased prevalence of diabetes and hypertension (Shah et al. 2022). In our study, NCD and osteoarthritis had higher impact on men’s HGS and less so among women. In line with this weaker association among women, Cheung et al. (2013) reported HGS to be associated with multiple chronic diseases and multimorbidity in men and women in Hong Kong, and the negative impact of multiple number of chronic diseases was particularly pronounced in men.

### Strengths and limitations

To our knowledge, this is the largest study (*n* = 7824) that provides HGS reference values with percentiles for the community-dwelling adult population stratified on disease status and sex. The HGS measurements were performed in the same location by trained healthcare professionals following a standardized protocol and reporting of HGS follows the Southampton protocol (Roberts et al. 2011), enabling comparison between studies and cohorts. The large number of participants ensures precise estimation of the reference values and is key in establishing references values for the sub-cohorts. Non-response bias by age, sex and education was corrected using full Tromsø population data, using national registry data for Tromsø without missing values. Another strength is the use of a statistical technique allowing for nonlinear association between age and HGS. The study has some limitations. First, participants agreeing to HGS testing might be healthier than non-participants and bias the HGS results upward, even though our sample was weighted and regarded representative to the general community-dwelling population. Secondly, the participants’ disease status was self-reported, and we had no objective test verifying whether NCD and osteoarthritis were present or not, which might misplace some respondents and dilute the true difference between the disease-free population and the disease groups.

## Conclusion

We provide up-to-date reference values for HGS, measured with a Jamar+ digital dynamometer, for a population-based community-dwelling adult population aged 40–84 years in the northern part of Norway. HGS was inversely associated with age and positively associated with body height. Men and women with non-communicable diseases had lower HGS than those without these conditions, while osteoarthritis was associated with lower HGS only among men.

## Data Availability

The data supporting the conclusions of this article are available at www.tromsoundersokelsen.no.

## Abbreviations

HGS: Hand grip strength
SD: Standard deviation
WHO: World Health Organization
NCD: Non-communicable diseases
SE: Standard error
CVD: Cardiovascular disease
CMD: Cardiometabolic disorders
OA: Osteoarthritis
